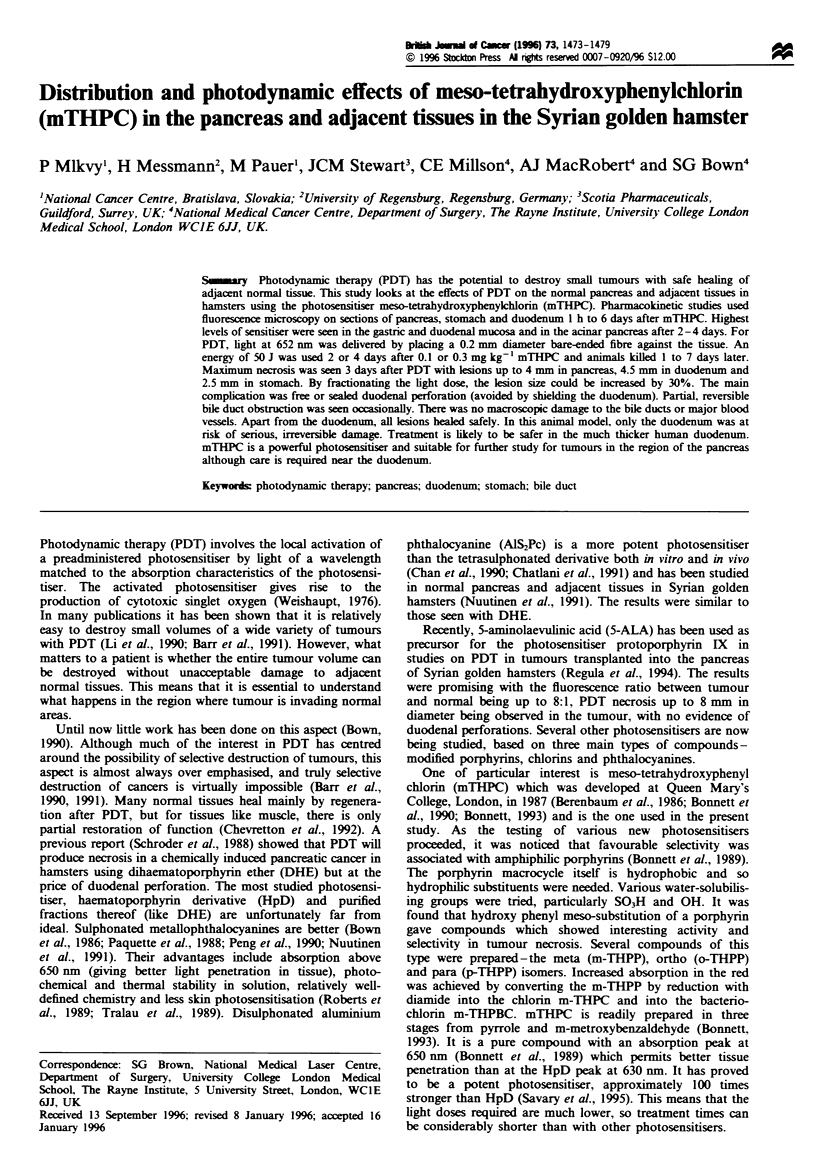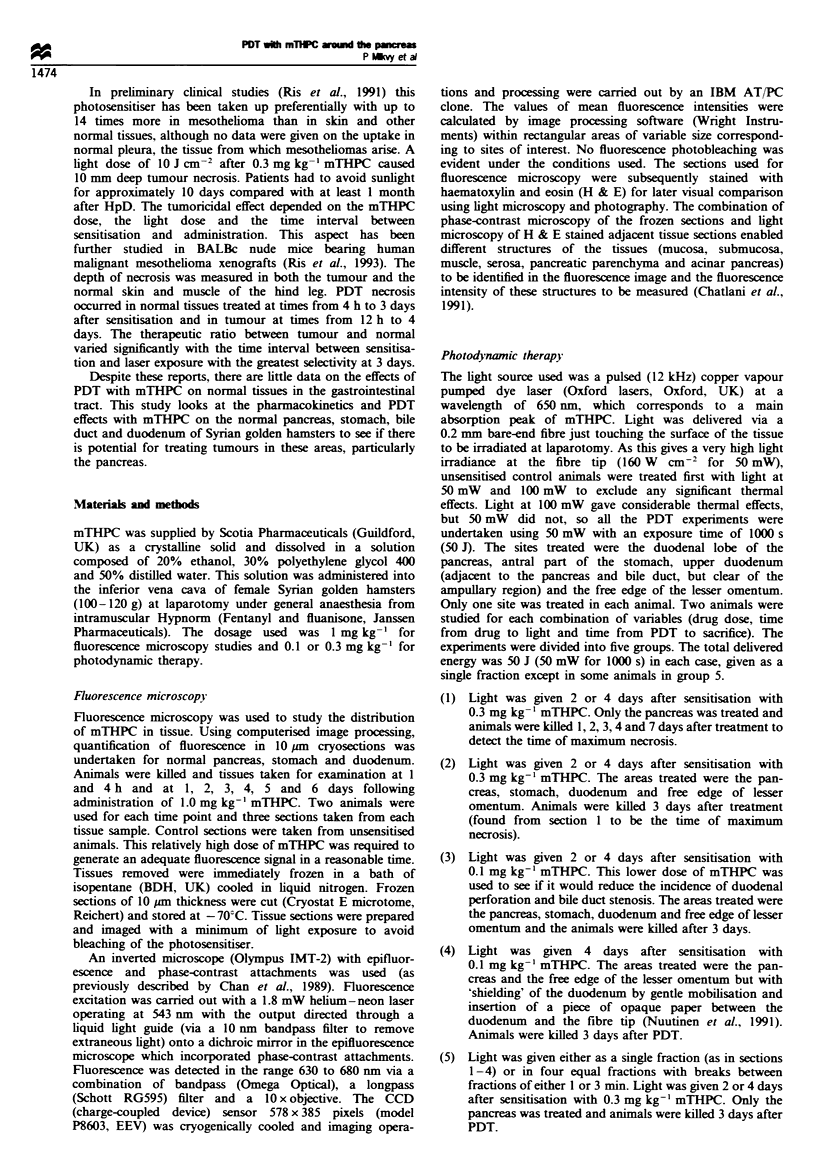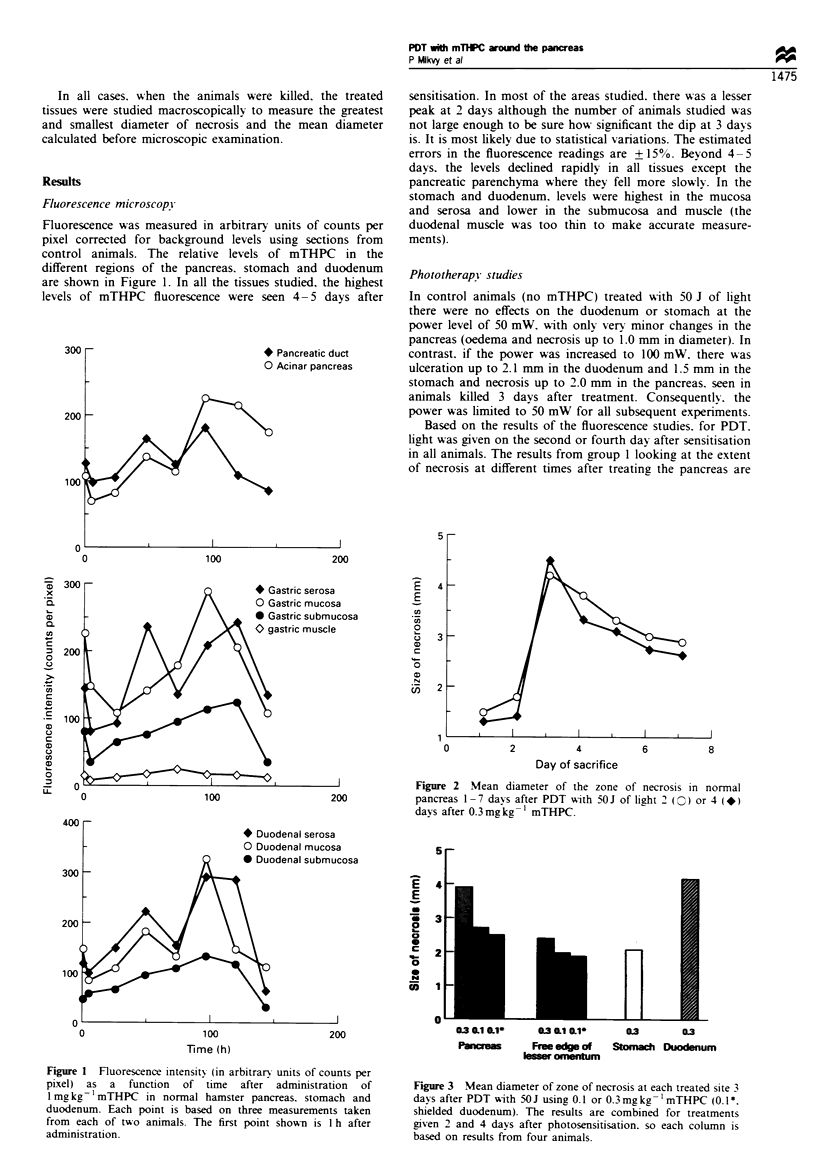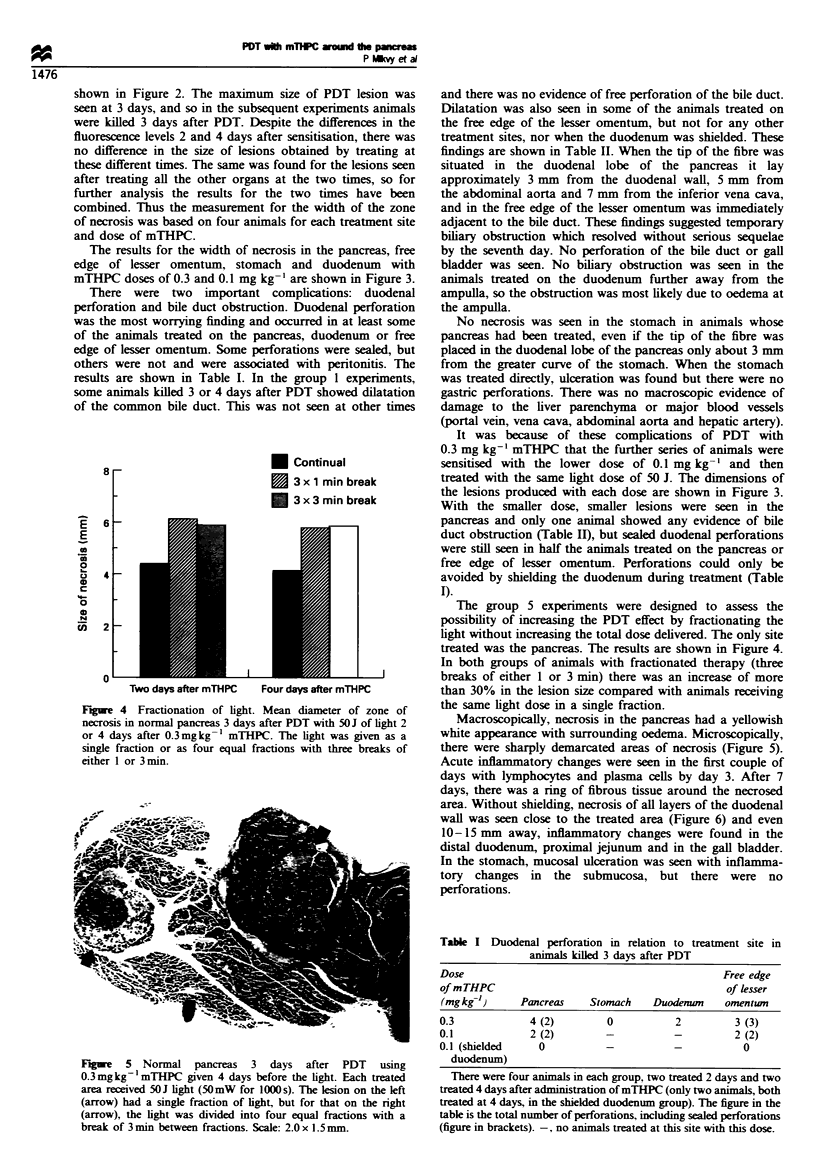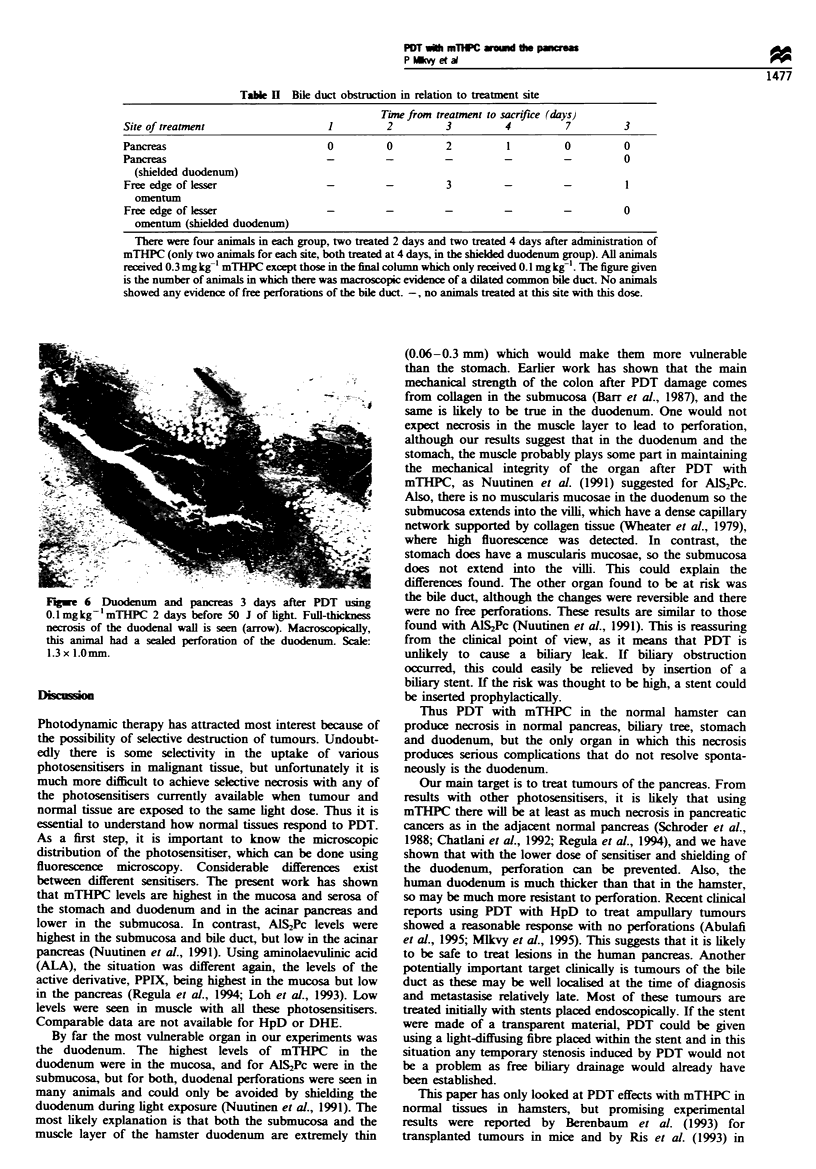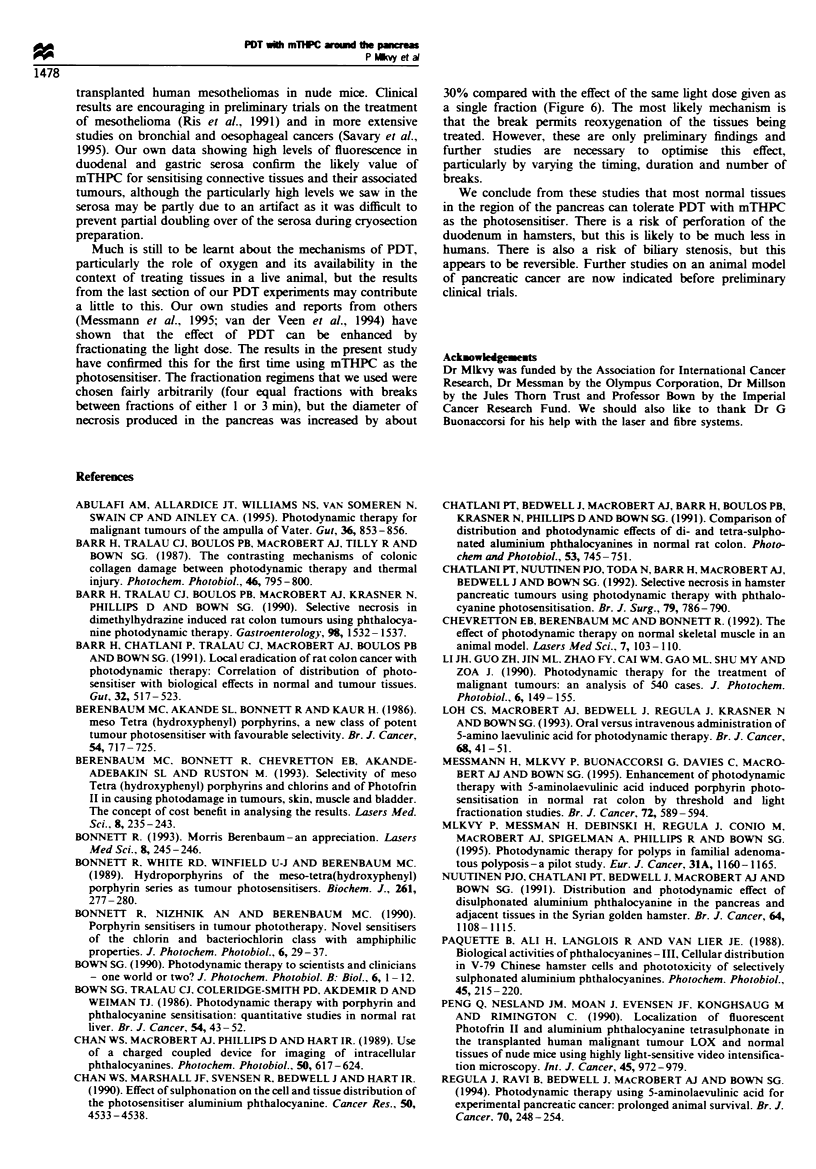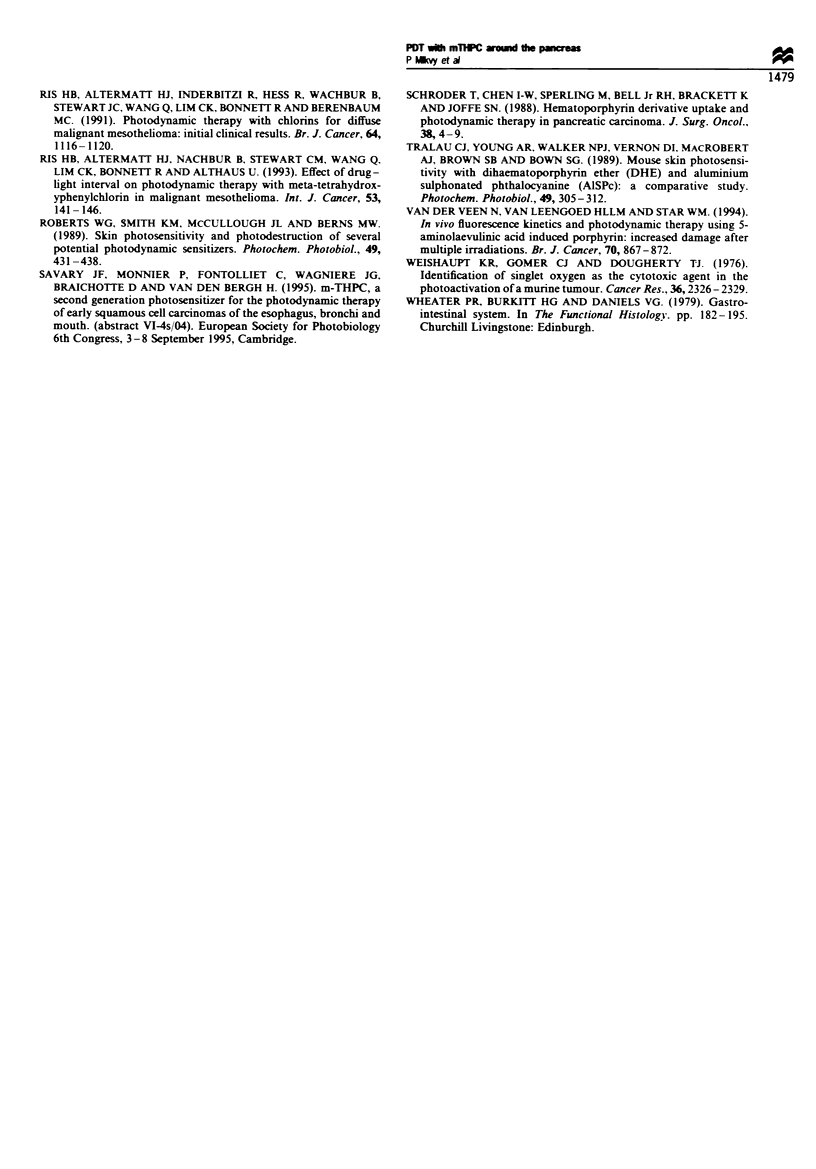# Distribution and photodynamic effects of meso-tetrahydroxyphenylchlorin (mTHPC) in the pancreas and adjacent tissues in the Syrian golden hamster.

**DOI:** 10.1038/bjc.1996.279

**Published:** 1996-06

**Authors:** P. Mlkvy, H. Messmann, M. Pauer, J. C. Stewart, C. E. Millson, A. J. MacRobert, S. G. Bown

**Affiliations:** National Cancer Centre, Bratislava, Slovakia.

## Abstract

**Images:**


British Journal of Cancer (1996) 73, 1473-1479

© 1996 Stockton Press All rights reserved 0007-0920/96 $12.00

### Distribution and photodynamic effects of meso-tetrahydroxyphenylchlorin (mTHPC) in the pancreas and adjacent tissues in the Syrian golden hamster

P Mlkvy[1], H Messmann[2], M Pauer[1], JCM Stewart[3], CE Millson[4], AJ MacRobert[4] and SG Bown[4]

[1]*National Cancer Centre, Bratislava, Slovakia;* [2]*University of Regensburg, Regensburg, Germany;* [3]*Scotia Pharmaceuticals, Guildford, Surrey, UK;* [4]*National Medical Cancer Centre, Department of Surgery, The Rayne Institute, University College London Medical School, London WC1E 6JJ, UK.*

**Summary** Photodynamic therapy (PDT) has the potential to destroy small tumours with safe healing of adjacent normal tissue. This study looks at the effects of PDT on the normal pancreas and adjacent tissues in hamsters using the photosensitiser meso-tetrahydroxyphenylchlorin (mTHPC). Pharmacokinetic studies used fluorescence microscopy on sections of pancreas, stomach and duodenum 1 h to 6 days after mTHPC. Highest levels of sensitiser were seen in the gastric and duodenal mucosa and in the acinar pancreas after 2-4 days. For PDT, light at 652 nm was delivered by placing a 0.2 mm diameter bare-ended fibre against the tissue. An energy of 50 J was used 2 or 4 days after 0.1 or 0.3 mg $kg^{-1}$ mTHPC and animals killed 1 to 7 days later. Maximum necrosis was seen 3 days after PDT with lesions up to 4 mm in pancreas, 4.5 mm in duodenum and 2.5 mm in stomach. By fractionating the light dose, the lesion size could be increased by 30%. The main complication was free or sealed duodenal perforation (avoided by shielding the duodenum). Partial, reversible bile duct obstruction was seen occasionally. There was no macroscopic damage to the bile ducts or major blood vessels. Apart from the duodenum, all lesions healed safely. In this animal model, only the duodenum was at risk of serious, irreversible damage. Treatment is likely to be safer in the much thicker human duodenum. mTHPC is a powerful photosensitiser and suitable for further study for tumours in the region of the pancreas although care is required near the duodenum.

**Keywords:** photodynamic therapy; pancreas; duodenum; stomach; bile duct

Photodynamic therapy (PDT) involves the local activation of a preadministered photosensitiser by light of a wavelength matched to the absorption characteristics of the photosensitiser. The activated photosensitiser gives rise to the production of cytotoxic singlet oxygen (Weishaupt, 1976). In many publications it has been shown that it is relatively easy to destroy small volumes of a wide variety of tumours with PDT (Li *et al.*, 1990; Barr *et al.*, 1991). However, what matters to a patient is whether the entire tumour volume can be destroyed without unacceptable damage to adjacent normal tissues. This means that it is essential to understand what happens in the region where tumour is invading normal areas.

Until now little work has been done on this aspect (Bown, 1990). Although much of the interest in PDT has centred around the possibility of selective destruction of tumours, this aspect is almost always over emphasised, and truly selective destruction of cancers is virtually impossible (Barr *et al.*, 1990, 1991). Many normal tissues heal mainly by regeneration after PDT, but for tissues like muscle, there is only partial restoration of function (Chevretton *et al.*, 1992). A previous report (Schroder *et al.*, 1988) showed that PDT will produce necrosis in a chemically induced pancreatic cancer in hamsters using dihaematoporphyrin ether (DHE) but at the price of duodenal perforation. The most studied photosensitiser, haematoporphyrin derivative (HpD) and purified fractions thereof (like DHE) are unfortunately far from ideal. Sulphonated metallophthalocyanines are better (Bown *et al.*, 1986; Paquette *et al.*, 1988; Peng *et al.*, 1990; Nuutinen *et al.*, 1991). Their advantages include absorption above 650 nm (giving better light penetration in tissue), photochemical and thermal stability in solution, relatively well-defined chemistry and less skin photosensitisation (Roberts *et al.*, 1989; Tralau *et al.*, 1989). Disulphonated aluminium phthalocyanine ($AlS_2Pc$) is a more potent photosensitiser than the tetrasulphonated derivative both *in vitro* and *in vivo* (Chan *et al.*, 1990; Chatlani *et al.*, 1991) and has been studied in normal pancreas and adjacent tissues in Syrian golden hamsters (Nuutinen *et al.*, 1991). The results were similar to those seen with DHE.

Recently, 5-aminolaevulinic acid (5-ALA) has been used as precursor for the photosensitiser protoporphyrin IX in studies on PDT in tumours transplanted into the pancreas of Syrian golden hamsters (Regula *et al.*, 1994). The results were promising with the fluorescence ratio between tumour and normal being up to 8:1, PDT necrosis up to 8 mm in diameter being observed in the tumour, with no evidence of duodenal perforations. Several other photosensitisers are now being studied, based on three main types of compounds - modified porphyrins, chlorins and phthalocyanines.

One of particular interest is meso-tetrahydroxyphenyl chlorin (mTHPC) which was developed at Queen Mary's College, London, in 1987 (Berenbaum *et al.*, 1986; Bonnett *et al.*, 1990; Bonnett, 1993) and is the one used in the present study. As the testing of various new photosensitisers proceeded, it was noticed that favourable selectivity was associated with amphiphilic porphyrins (Bonnett *et al.*, 1989). The porphyrin macrocycle itself is hydrophobic and so hydrophilic substituents were needed. Various water-solubilising groups were tried, particularly $SO_3H$ and OH. It was found that hydroxy phenyl meso-substitution of a porphyrin gave compounds which showed interesting activity and selectivity in tumour necrosis. Several compounds of this type were prepared - the meta (m-THPP), ortho (o-THPP) and para (p-THPP) isomers. Increased absorption in the red was achieved by converting the m-THPP by reduction with diamide into the chlorin m-THPC and into the bacteriochlorin m-THPBC. mTHPC is readily prepared in three stages from pyrrole and m-metroxybenzaldehyde (Bonnett, 1993). It is a pure compound with an absorption peak at 650 nm (Bonnett *et al.*, 1989) which permits better tissue penetration than at the HpD peak at 630 nm. It has proved to be a potent photosensitiser, approximately 100 times stronger than HpD (Savary *et al.*, 1995). This means that the light doses required are much lower, so treatment times can be considerably shorter than with other photosensitisers.

Correspondence: SG Brown, National Medical Laser Centre, Department of Surgery, University College London Medical School, The Rayne Institute, 5 University Street, London, WC1E 6JJ, UK
Received 13 September 1996; revised 8 January 1996; accepted 16 January 1996

In preliminary clinical studies (Ris *et al.*, 1991) this photosensitiser has been taken up preferentially with up to 14 times more in mesothelioma than in skin and other normal tissues, although no data were given on the uptake in normal pleura, the tissue from which mesotheliomas arise. A light dose of 10 J $cm^{-2}$ after 0.3 mg $kg^{-1}$ mTHPC caused 10 mm deep tumour necrosis. Patients had to avoid sunlight for approximately 10 days compared with at least 1 month after HpD. The tumoricidal effect depended on the mTHPC dose, the light dose and the time interval between sensitisation and administration. This aspect has been further studied in BALBc nude mice bearing human malignant mesothelioma xenografts (Ris *et al.*, 1993). The depth of necrosis was measured in both the tumour and the normal skin and muscle of the hind leg. PDT necrosis occurred in normal tissues treated at times from 4 h to 3 days after sensitisation and in tumour at times from 12 h to 4 days. The therapeutic ratio between tumour and normal varied significantly with the time interval between sensitisation and laser exposure with the greatest selectivity at 3 days.

Despite these reports, there are little data on the effects of PDT with mTHPC on normal tissues in the gastrointestinal tract. This study looks at the pharmacokinetics and PDT effects with mTHPC on the normal pancreas, stomach, bile duct and duodenum of Syrian golden hamsters to see if there is potential for treating tumours in these areas, particularly the pancreas.

### Materials and methods mTHPC was supplied by Scotia Pharmaceuticals (Guildford, UK) as a crystalline solid and dissolved in a solution composed of 20% ethanol, 30% polyethylene glycol 400 and 50% distilled water. This solution was administered into the inferior vena cava of female Syrian golden hamsters (100–120 g) at laparotomy under general anaesthesia from intramuscular Hypnorm (Fentanyl and fluanisone, Janssen Pharmaceuticals). The dosage used was 1 mg $kg^{-1}$ for fluorescence microscopy studies and 0.1 or 0.3 mg $kg^{-1}$ for photodynamic therapy.

### *Fluorescence microscopy*

Fluorescence microscopy was used to study the distribution of mTHPC in tissue. Using computerised image processing, quantification of fluorescence in 10 $\mu m$ cryosections was undertaken for normal pancreas, stomach and duodenum. Animals were killed and tissues taken for examination at 1 and 4 h and at 1, 2, 3, 4, 5 and 6 days following administration of 1.0 mg $kg^{-1}$ mTHPC. Two animals were used for each time point and three sections taken from each tissue sample. Control sections were taken from unsensitised animals. This relatively high dose of mTHPC was required to generate an adequate fluorescence signal in a reasonable time. Tissues removed were immediately frozen in a bath of isopentane (BDH, UK) cooled in liquid nitrogen. Frozen sections of 10 $\mu m$ thickness were cut (Cryostat E microtome, Reichert) and stored at −70°C. Tissue sections were prepared and imaged with a minimum of light exposure to avoid bleaching of the photosensitiser.

An inverted microscope (Olympus IMT-2) with epifluorescence and phase-contrast attachments was used (as previously described by Chan *et al.*, 1989). Fluorescence excitation was carried out with a 1.8 mW helium–neon laser operating at 543 nm with the output directed through a liquid light guide (via a 10 nm bandpass filter to remove extraneous light) onto a dichroic mirror in the epifluorescence microscope which incorporated phase-contrast attachments. Fluorescence was detected in the range 630 to 680 nm via a combination of bandpass (Omega Optical), a longpass (Schott RG595) filter and a 10 × objective. The CCD (charge-coupled device) sensor 578 × 385 pixels (model P8603, EEV) was cryogenically cooled and imaging operations and processing were carried out by an IBM AT/PC clone. The values of mean fluorescence intensities were calculated by image processing software (Wright Instruments) within rectangular areas of variable size corresponding to sites of interest. No fluorescence photobleaching was evident under the conditions used. The sections used for fluorescence microscopy were subsequently stained with haematoxylin and eosin (H & E) for later visual comparison using light microscopy and photography. The combination of phase-contrast microscopy of the frozen sections and light microscopy of H & E stained adjacent tissue sections enabled different structures of the tissues (mucosa, submucosa, muscle, serosa, pancreatic parenchyma and acinar pancreas) to be identified in the fluorescence image and the fluorescence intensity of these structures to be measured (Chatlani *et al.*, 1991).

### *Photodynamic therapy*

The light source used was a pulsed (12 kHz) copper vapour pumped dye laser (Oxford lasers, Oxford, UK) at a wavelength of 650 nm, which corresponds to a main absorption peak of mTHPC. Light was delivered via a 0.2 mm bare-end fibre just touching the surface of the tissue to be irradiated at laparotomy. As this gives a very high light irradiance at the fibre tip (160 W $cm^{-2}$ for 50 mW), unsensitised control animals were treated first with light at 50 mW and 100 mW to exclude any significant thermal effects. Light at 100 mW gave considerable thermal effects, but 50 mW did not, so all the PDT experiments were undertaken using 50 mW with an exposure time of 1000 s (50 J). The sites treated were the duodenal lobe of the pancreas, antral part of the stomach, upper duodenum (adjacent to the pancreas and bile duct, but clear of the ampullary region) and the free edge of the lesser omentum. Only one site was treated in each animal. Two animals were studied for each combination of variables (drug dose, time from drug to light and time from PDT to sacrifice). The experiments were divided into five groups. The total delivered energy was 50 J (50 mW for 1000 s) in each case, given as a single fraction except in some animals in group 5.

(1) Light was given 2 or 4 days after sensitisation with 0.3 mg $kg^{-1}$ mTHPC. Only the pancreas was treated and animals were killed 1, 2, 3, 4 and 7 days after treatment to detect the time of maximum necrosis.

(2) Light was given 2 or 4 days after sensitisation with 0.3 mg $kg^{-1}$ mTHPC. The areas treated were the pancreas, stomach, duodenum and free edge of lesser omentum. Animals were killed 3 days after treatment (found from section 1 to be the time of maximum necrosis).

(3) Light was given 2 or 4 days after sensitisation with 0.1 mg $kg^{-1}$ mTHPC. This lower dose of mTHPC was used to see if it would reduce the incidence of duodenal perforation and bile duct stenosis. The areas treated were the pancreas, stomach, duodenum and free edge of lesser omentum and the animals were killed after 3 days.

(4) Light was given 4 days after sensitisation with 0.1 mg $kg^{-1}$ mTHPC. The areas treated were the pancreas and the free edge of the lesser omentum but with 'shielding' of the duodenum by gentle mobilisation and insertion of a piece of opaque paper between the duodenum and the fibre tip (Nuutinen *et al.*, 1991). Animals were killed 3 days after PDT.

(5) Light was given either as a single fraction (as in sections 1–4) or in four equal fractions with breaks between fractions of either 1 or 3 min. Light was given 2 or 4 days after sensitisation with 0.3 mg $kg^{-1}$ mTHPC. Only the pancreas was treated and animals were killed 3 days after PDT.

In all cases, when the animals were killed, the treated tissues were studied macroscopically to measure the greatest and smallest diameter of necrosis and the mean diameter calculated before microscopic examination.

### Results

### *Fluorescence microscopy*

Fluorescence was measured in arbitrary units of counts per pixel corrected for background levels using sections from control animals. The relative levels of mTHPC in the different regions of the pancreas, stomach and duodenum are shown in Figure 1. In all the tissues studied, the highest levels of mTHPC fluorescence were seen 4–5 days after sensitisation. In most of the areas studied, there was a lesser peak at 2 days although the number of animals studied was not large enough to be sure how significant the dip at 3 days is. It is most likely due to statistical variations. The estimated errors in the fluorescence readings are $\pm 15\%$. Beyond 4–5 days, the levels declined rapidly in all tissues except the pancreatic parenchyma where they fell more slowly. In the stomach and duodenum, levels were highest in the mucosa and serosa and lower in the submucosa and muscle (the duodenal muscle was too thin to make accurate measurements).

**Figure 1** Fluorescence intensity (in arbitrary units of counts per pixel) as a function of time after administration of $1\,mg\,kg^{-1}$ mTHPC in normal hamster pancreas, stomach and duodenum. Each point is based on three measurements taken from each of two animals. The first point shown is 1 h after administration.

### *Phototherapy studies*

In control animals (no mTHPC) treated with 50 J of light there were no effects on the duodenum or stomach at the power level of 50 mW, with only very minor changes in the pancreas (oedema and necrosis up to 1.0 mm in diameter). In contrast, if the power was increased to 100 mW, there was ulceration up to 2.1 mm in the duodenum and 1.5 mm in the stomach and necrosis up to 2.0 mm in the pancreas, seen in animals killed 3 days after treatment. Consequently, the power was limited to 50 mW for all subsequent experiments.

Based on the results of the fluorescence studies, for PDT, light was given on the second or fourth day after sensitisation in all animals. The results from group 1 looking at the extent of necrosis at different times after treating the pancreas are

**Figure 2** Mean diameter of the zone of necrosis in normal pancreas 1–7 days after PDT with 50 J of light 2 (○) or 4 (◆) days after $0.3\,mg\,kg^{-1}$ mTHPC.

**Figure 3** Mean diameter of zone of necrosis at each treated site 3 days after PDT with 50 J using 0.1 or $0.3\,mg\,kg^{-1}$ mTHPC (0.1*, shielded duodenum). The results are combined for treatments given 2 and 4 days after photosensitisation, so each column is based on results from four animals.

shown in Figure 2. The maximum size of PDT lesion was seen at 3 days, and so in the subsequent experiments animals were killed 3 days after PDT. Despite the differences in the fluorescence levels 2 and 4 days after sensitisation, there was no difference in the size of lesions obtained by treating at these different times. The same was found for the lesions seen after treating all the other organs at the two times, so for further analysis the results for the two times have been combined. Thus the measurement for the width of the zone of necrosis was based on four animals for each treatment site and dose of mTHPC.

The results for the width of necrosis in the pancreas, free edge of lesser omentum, stomach and duodenum with mTHPC doses of 0.3 and 0.1 mg $kg^{-1}$ are shown in Figure 3.

There were two important complications: duodenal perforation and bile duct obstruction. Duodenal perforation was the most worrying finding and occurred in at least some of the animals treated on the pancreas, duodenum or free edge of lesser omentum. Some perforations were sealed, but others were not and were associated with peritonitis. The results are shown in Table I. In the group 1 experiments, some animals killed 3 or 4 days after PDT showed dilatation of the common bile duct. This was not seen at other times

**Figure 4** Fractionation of light. Mean diameter of zone of necrosis in normal pancreas 3 days after PDT with 50 J of light 2 or 4 days after 0.3 mg $kg^{-1}$ mTHPC. The light was given as a single fraction or as four equal fractions with three breaks of either 1 or 3 min.

**Figure 5** Normal pancreas 3 days after PDT using 0.3 mg $kg^{-1}$ mTHPC given 4 days before the light. Each treated area received 50 J light (50 mW for 1000 s). The lesion on the left (arrow) had a single fraction of light, but for that on the right (arrow), the light was divided into four equal fractions with a break of 3 min between fractions. Scale: 2.0 × 1.5 mm.

and there was no evidence of free perforation of the bile duct. Dilatation was also seen in some of the animals treated on the free edge of the lesser omentum, but not for any other treatment sites, nor when the duodenum was shielded. These findings are shown in Table II. When the tip of the fibre was situated in the duodenal lobe of the pancreas it lay approximately 3 mm from the duodenal wall, 5 mm from the abdominal aorta and 7 mm from the inferior vena cava, and in the free edge of the lesser omentum was immediately adjacent to the bile duct. These findings suggested temporary biliary obstruction which resolved without serious sequelae by the seventh day. No perforation of the bile duct or gall bladder was seen. No biliary obstruction was seen in the animals treated on the duodenum further away from the ampulla, so the obstruction was most likely due to oedema at the ampulla.

No necrosis was seen in the stomach in animals whose pancreas had been treated, even if the tip of the fibre was placed in the duodenal lobe of the pancreas only about 3 mm from the greater curve of the stomach. When the stomach was treated directly, ulceration was found but there were no gastric perforations. There was no macroscopic evidence of damage to the liver parenchyma or major blood vessels (portal vein, vena cava, abdominal aorta and hepatic artery).

It was because of these complications of PDT with 0.3 mg $kg^{-1}$ mTHPC that the further series of animals were sensitised with the lower dose of 0.1 mg $kg^{-1}$ and then treated with the same light dose of 50 J. The dimensions of the lesions produced with each dose are shown in Figure 3. With the smaller dose, smaller lesions were seen in the pancreas and only one animal showed any evidence of bile duct obstruction (Table II), but sealed duodenal perforations were still seen in half the animals treated on the pancreas or free edge of lesser omentum. Perforations could only be avoided by shielding the duodenum during treatment (Table I).

The group 5 experiments were designed to assess the possibility of increasing the PDT effect by fractionating the light without increasing the total dose delivered. The only site treated was the pancreas. The results are shown in Figure 4. In both groups of animals with fractionated therapy (three breaks of either 1 or 3 min) there was an increase of more than 30% in the lesion size compared with animals receiving the same light dose in a single fraction.

Macroscopically, necrosis in the pancreas had a yellowish white appearance with surrounding oedema. Microscopically, there were sharply demarcated areas of necrosis (Figure 5). Acute inflammatory changes were seen in the first couple of days with lymphocytes and plasma cells by day 3. After 7 days, there was a ring of fibrous tissue around the necrosed area. Without shielding, necrosis of all layers of the duodenal wall was seen close to the treated area (Figure 6) and even 10–15 mm away, inflammatory changes were found in the distal duodenum, proximal jejunum and in the gall bladder. In the stomach, mucosal ulceration was seen with inflammatory changes in the submucosa, but there were no perforations.

**Table I** Duodenal perforation in relation to treatment site in animals killed 3 days after PDT

| *Dose of mTHPC (mg $kg^{-1}$)* | *Pancreas* | *Stomach* | *Duodenum* | *Free edge of lesser omentum* |
|---|---|---|---|---|
| 0.3 | 4 (2) | 0 | 2 | 3 (3) |
| 0.1 | 2 (2) | – | – | 2 (2) |
| 0.1 (shielded duodenum) | 0 | – | – | 0 |

There were four animals in each group, two treated 2 days and two treated 4 days after administration of mTHPC (only two animals, both treated at 4 days, in the shielded duodenum group). The figure in the table is the total number of perforations, including sealed perforations (figure in brackets). –, no animals treated at this site with this dose.

**Table II** Bile duct obstruction in relation to treatment site

| Site of treatment | *Time from treatment to sacrifice (days)* 1 | 2 | 3 | 4 | 7 | 3 |
|---|---|---|---|---|---|---|
| Pancreas | 0 | 0 | 2 | 1 | 0 | 0 |
| Pancreas (shielded duodenum) | – | – | – | – | – | 0 |
| Free edge of lesser omentum | – | – | 3 | – | – | 1 |
| Free edge of lesser omentum (shielded duodenum) | – | – | – | – | – | 0 |

There were four animals in each group, two treated 2 days and two treated 4 days after administration of mTHPC (only two animals for each site, both treated at 4 days, in the shielded duodenum group). All animals received 0.3 mg $kg^{-1}$ mTHPC except those in the final column which only received 0.1 mg $kg^{-1}$. The figure given is the number of animals in which there was macroscopic evidence of a dilated common bile duct. No animals showed any evidence of free perforations of the bile duct. –, no animals treated at this site with this dose.

**Figure 6** Duodenum and pancreas 3 days after PDT using 0.1 mg $kg^{-1}$ mTHPC 2 days before 50 J of light. Full-thickness necrosis of the duodenal wall is seen (arrow). Macroscopically, this animal had a sealed perforation of the duodenum. Scale: 1.3 × 1.0 mm.

### Discussion

Photodynamic therapy has attracted most interest because of the possibility of selective destruction of tumours. Undoubtedly there is some selectivity in the uptake of various photosensitisers in malignant tissue, but unfortunately it is much more difficult to achieve selective necrosis with any of the photosensitisers currently available when tumour and normal tissue are exposed to the same light dose. Thus it is essential to understand how normal tissues respond to PDT. As a first step, it is important to know the microscopic distribution of the photosensitiser, which can be done using fluorescence microscopy. Considerable differences exist between different sensitisers. The present work has shown that mTHPC levels are highest in the mucosa and serosa of the stomach and duodenum and in the acinar pancreas and lower in the submucosa. In contrast, $AlS_2Pc$ levels were highest in the submucosa and bile duct, but low in the acinar pancreas (Nuutinen *et al.*, 1991). Using aminolaevulinic acid (ALA), the situation was different again, the levels of the active derivative, PPIX, being highest in the mucosa but low in the pancreas (Regula *et al.*, 1994; Loh *et al.*, 1993). Low levels were seen in muscle with all these photosensitisers. Comparable data are not available for HpD or DHE.

By far the most vulnerable organ in our experiments was the duodenum. The highest levels of mTHPC in the duodenum were in the mucosa, and for $AlS_2Pc$ were in the submucosa, but for both, duodenal perforations were seen in many animals and could only be avoided by shielding the duodenum during light exposure (Nuutinen *et al.*, 1991). The most likely explanation is that both the submucosa and the muscle layer of the hamster duodenum are extremely thin (0.06–0.3 mm) which would make them more vulnerable than the stomach. Earlier work has shown that the main mechanical strength of the colon after PDT damage comes from collagen in the submucosa (Barr *et al.*, 1987), and the same is likely to be true in the duodenum. One would not expect necrosis in the muscle layer to lead to perforation, although our results suggest that in the duodenum and the stomach, the muscle probably plays some part in maintaining the mechanical integrity of the organ after PDT with mTHPC, as Nuutinen *et al.* (1991) suggested for $AlS_2Pc$. Also, there is no muscularis mucosae in the duodenum so the submucosa extends into the villi, which have a dense capillary network supported by collagen tissue (Wheater *et al.*, 1979), where high fluorescence was detected. In contrast, the stomach does have a muscularis mucosae, so the submucosa does not extend into the villi. This could explain the differences found. The other organ found to be at risk was the bile duct, although the changes were reversible and there were no free perforations. These results are similar to those found with $AlS_2Pc$ (Nuutinen *et al.*, 1991). This is reassuring from the clinical point of view, as it means that PDT is unlikely to cause a biliary leak. If biliary obstruction occurred, this could easily be relieved by insertion of a biliary stent. If the risk was thought to be high, a stent could be inserted prophylactically.

Thus PDT with mTHPC in the normal hamster can produce necrosis in normal pancreas, biliary tree, stomach and duodenum, but the only organ in which this necrosis produces serious complications that do not resolve spontaneously is the duodenum.

Our main target is to treat tumours of the pancreas. From results with other photosensitisers, it is likely that using mTHPC there will be at least as much necrosis in pancreatic cancers as in the adjacent normal pancreas (Schroder *et al.*, 1988; Chatlani *et al.*, 1992; Regula *et al.*, 1994), and we have shown that with the lower dose of sensitiser and shielding of the duodenum, perforation can be prevented. Also, the human duodenum is much thicker than that in the hamster, so may be much more resistant to perforation. Recent clinical reports using PDT with HpD to treat ampullary tumours showed a reasonable response with no perforations (Abulafi *et al.*, 1995; Mlkvy *et al.*, 1995). This suggests that it is likely to be safe to treat lesions in the human pancreas. Another potentially important target clinically is tumours of the bile duct as these may be well localised at the time of diagnosis and metastasise relatively late. Most of these tumours are treated initially with stents placed endoscopically. If the stent were made of a transparent material, PDT could be given using a light-diffusing fibre placed within the stent and in this situation any temporary stenosis induced by PDT would not be a problem as free biliary drainage would already have been established.

This paper has only looked at PDT effects with mTHPC in normal tissues in hamsters, but promising experimental results were reported by Berenbaum *et al.* (1993) for transplanted tumours in mice and by Ris *et al.* (1993) in transplanted human mesotheliomas in nude mice. Clinical results are encouraging in preliminary trials on the treatment of mesothelioma (Ris *et al.*, 1991) and in more extensive studies on bronchial and oesophageal cancers (Savary *et al.*, 1995). Our own data showing high levels of fluorescence in duodenal and gastric serosa confirm the likely value of mTHPC for sensitising connective tissues and their associated tumours, although the particularly high levels we saw in the serosa may be partly due to an artifact as it was difficult to prevent partial doubling over of the serosa during cryosection preparation.

Much is still to be learnt about the mechanisms of PDT, particularly the role of oxygen and its availability in the context of treating tissues in a live animal, but the results from the last section of our PDT experiments may contribute a little to this. Our own studies and reports from others (Messmann *et al.*, 1995; van der Veen *et al.*, 1994) have shown that the effect of PDT can be enhanced by fractionating the light dose. The results in the present study have confirmed this for the first time using mTHPC as the photosensitiser. The fractionation regimens that we used were chosen fairly arbitrarily (four equal fractions with breaks between fractions of either 1 or 3 min), but the diameter of necrosis produced in the pancreas was increased by about 30% compared with the effect of the same light dose given as a single fraction (Figure 6). The most likely mechanism is that the break permits reoxygenation of the tissues being treated. However, these are only preliminary findings and further studies are necessary to optimise this effect, particularly by varying the timing, duration and number of breaks.

We conclude from these studies that most normal tissues in the region of the pancreas can tolerate PDT with mTHPC as the photosensitiser. There is a risk of perforation of the duodenum in hamsters, but this is likely to be much less in humans. There is also a risk of biliary stenosis, but this appears to be reversible. Further studies on an animal model of pancreatic cancer are now indicated before preliminary clinical trials.

**Acknowledgements**

Dr Mlkvy was funded by the Association for International Cancer Research, Dr Messman by the Olympus Corporation, Dr Millson by the Jules Thorn Trust and Professor Bown by the Imperial Cancer Research Fund. We should also like to thank Dr G Buonaccorsi for his help with the laser and fibre systems.

### References

ABULAFI AM, ALLARDICE JT, WILLIAMS NS, VAN SOMEREN N, SWAIN CP AND AINLEY CA. (1995). Photodynamic therapy for malignant tumours of the ampulla of Vater. *Gut*, **36**, 853–856.

BARR H, TRALAU CJ, BOULOS PB, MACROBERT AJ, TILLY R AND BOWN SG. (1987). The contrasting mechanisms of colonic collagen damage between photodynamic therapy and thermal injury. *Photochem. Photobiol.*, **46**, 795–800.

BARR H, TRALAU CJ, BOULOS PB, MACROBERT AJ, KRASNER N, PHILLIPS D AND BOWN SG. (1990). Selective necrosis in dimethylhydrazine induced rat colon tumours using phthalocyanine photodynamic therapy. *Gastroenterology*, **98**, 1532–1537.

BARR H, CHATLANI P, TRALAU CJ, MACROBERT AJ, BOULOS PB AND BOWN SG. (1991). Local eradication of rat colon cancer with photodynamic therapy: Correlation of distribution of photosensitiser with biological effects in normal and tumour tissues. *Gut*, **32**, 517–523.

BERENBAUM MC, AKANDE SL, BONNETT R AND KAUR H. (1986). meso Tetra (hydroxyphenyl) porphyrins, a new class of potent tumour photosensitiser with favourable selectivity. *Br. J. Cancer*, **54**, 717–725.

BERENBAUM MC, BONNETT R, CHEVRETTON EB, AKANDE-ADEBAKIN SL AND RUSTON M. (1993). Selectivity of meso Tetra (hydroxyphenyl) porphyrins and chlorins and of Photofrin II in causing photodamage in tumours, skin, muscle and bladder. The concept of cost benefit in analysing the results. *Lasers Med. Sci.*, **8**, 235–243.

BONNETT R. (1993). Morris Berenbaum–an appreciation. *Lasers Med Sci.*, **8**, 245–246.

BONNETT R, WHITE RD, WINFIELD U-J AND BERENBAUM MC. (1989). Hydroporphyrins of the meso-tetra(hydroxyphenyl) porphyrin series as tumour photosensitisers. *Biochem. J.*, **261**, 277–280.

BONNETT R, NIZHNIK AN AND BERENBAUM MC. (1990). Porphyrin sensitisers in tumour phototherapy. Novel sensitisers of the chlorin and bacteriochlorin class with amphiphilic properties. *J. Photochem. Photobiol.*, **6**, 29–37.

BOWN SG. (1990). Photodynamic therapy to scientists and clinicians – one world or two? *J. Photochem. Photobiol. B: Biol.*, **6**, 1–12.

BOWN SG, TRALAU CJ, COLERIDGE-SMITH PD, AKDEMIR D AND WEIMAN TJ. (1986). Photodynamic therapy with porphyrin and phthalocyanine sensitisation: quantitative studies in normal rat liver. *Br. J. Cancer*, **54**, 43–52.

CHAN WS, MACROBERT AJ, PHILLIPS D AND HART IR. (1989). Use of a charged coupled device for imaging of intracellular phthalocyanines. *Photochem. Photobiol.*, **50**, 617–624.

CHAN WS, MARSHALL JF, SVENSEN R, BEDWELL J AND HART IR. (1990). Effect of sulphonation on the cell and tissue distribution of the photosensitiser aluminium phthalocyanine. *Cancer Res.*, **50**, 4533–4538.

CHATLANI PT, BEDWELL J, MACROBERT AJ, BARR H, BOULOS PB, KRASNER N, PHILLIPS D AND BOWN SG. (1991). Comparison of distribution and photodynamic effects of di- and tetra-sulphonated aluminium phthalocyanines in normal rat colon. *Photochem and Photobiol.*, **53**, 745–751.

CHATLANI PT, NUUTINEN PJO, TODA N, BARR H, MACROBERT AJ, BEDWELL J AND BOWN SG. (1992). Selective necrosis in hamster pancreatic tumours using photodynamic therapy with phthalocyanine photosensitisation. *Br. J. Surg.*, **79**, 786–790.

CHEVRETTON EB, BERENBAUM MC AND BONNETT R. (1992). The effect of photodynamic therapy on normal skeletal muscle in an animal model. *Lasers Med Sci.*, **7**, 103–110.

LI JH, GUO ZH, JIN ML, ZHAO FY, CAI WM, GAO ML, SHU MY AND ZOA J. (1990). Photodynamic therapy for the treatment of malignant tumours: an analysis of 540 cases. *J. Photochem. Photobiol.*, **6**, 149–155.

LOH CS, MACROBERT AJ, BEDWELL J, REGULA J, KRASNER N AND BOWN SG. (1993). Oral versus intravenous administration of 5-amino laevulinic acid for photodynamic therapy. *Br. J. Cancer*, **68**, 41–51.

MESSMANN H, MLKVY P, BUONACCORSI G, DAVIES C, MACROBERT AJ AND BOWN SG. (1995). Enhancement of photodynamic therapy with 5-aminolaevulinic acid induced porphyrin photosensitisation in normal rat colon by threshold and light fractionation studies. *Br. J. Cancer*, **72**, 589–594.

MLKVY P, MESSMAN H, DEBINSKI H, REGULA J, CONIO M, MACROBERT AJ, SPIGELMAN A, PHILLIPS R AND BOWN SG. (1995). Photodynamic therapy for polyps in familial adenomatous polyposis–a pilot study. *Eur. J. Cancer*, **31A**, 1160–1165.

NUUTINEN PJO, CHATLANI PT, BEDWELL J, MACROBERT AJ AND BOWN SG. (1991). Distribution and photodynamic effect of disulphonated aluminium phthalocyanine in the pancreas and adjacent tissues in the Syrian golden hamster. *Br. J. Cancer*, **64**, 1108–1115.

PAQUETTE B, ALI H, LANGLOIS R AND VAN LIER JE. (1988). Biological activities of phthalocyanines – III, Cellular distribution in V-79 Chinese hamster cells and phototoxicity of selectively sulphonated aluminium phthalocyanines. *Photochem. Photobiol.*, **45**, 215–220.

PENG Q, NESLAND JM, MOAN J, EVENSEN JF, KONGHSAUG M AND RIMINGTON C. (1990). Localization of fluorescent Photofrin II and aluminium phthalocyanine tetrasulphonate in the transplanted human malignant tumour LOX and normal tissues of nude mice using highly light-sensitive video intensification microscopy. *Int. J. Cancer*, **45**, 972–979.

REGULA J, RAVI B, BEDWELL J, MACROBERT AJ AND BOWN SG. (1994). Photodynamic therapy using 5-aminolaevulinic acid for experimental pancreatic cancer: prolonged animal survival. *Br. J. Cancer*, **70**, 248–254.

RIS HB, ALTERMATT HJ, INDERBITZI R, HESS R, WACHBUR B, STEWART JC, WANG Q, LIM CK, BONNETT R AND BERENBAUM MC. (1991). Photodynamic therapy with chlorins for diffuse malignant mesothelioma: initial clinical results. *Br. J. Cancer*, **64**, 1116–1120.

RIS HB, ALTERMATT HJ, NACHBUR B, STEWART CM, WANG Q, LIM CK, BONNETT R AND ALTHAUS U. (1993). Effect of drug–light interval on photodynamic therapy with meta-tetrahydroxyphenylchlorin in malignant mesothelioma. *Int. J. Cancer*, **53**, 141–146.

ROBERTS WG, SMITH KM, McCULLOUGH JL AND BERNS MW. (1989). Skin photosensitivity and photodestruction of several potential photodynamic sensitizers. *Photochem. Photobiol.*, **49**, 431–438.

SAVARY JF, MONNIER P, FONTOLLIET C, WAGNIERE JG, BRAICHOTTE D AND VAN DEN BERGH H. (1995). m-THPC, a second generation photosensitizer for the photodynamic therapy of early squamous cell carcinomas of the esophagus, bronchi and mouth. (abstract VI-4s/04). European Society for Photobiology 6th Congress, 3–8 September 1995, Cambridge.

SCHRODER T, CHEN I-W, SPERLING M, BELL Jr RH, BRACKETT K AND JOFFE SN. (1988). Hematoporphyrin derivative uptake and photodynamic therapy in pancreatic carcinoma. *J. Surg. Oncol.*, **38**, 4–9.

TRALAU CJ, YOUNG AR, WALKER NPJ, VERNON DI, MACROBERT AJ, BROWN SB AND BOWN SG. (1989). Mouse skin photosensitivity with dihaematoporphyrin ether (DHE) and aluminium sulphonated phthalocyanine (AlSPc): a comparative study. *Photochem. Photobiol.*, **49**, 305–312.

VAN DER VEEN N, VAN LEENGOED HLLM AND STAR WM. (1994). *In vivo* fluorescence kinetics and photodynamic therapy using 5-aminolaevulinic acid induced porphyrin: increased damage after multiple irradiations. *Br. J. Cancer*, **70**, 867–872.

WEISHAUPT KR, GOMER CJ AND DOUGHERTY TJ. (1976). Identification of singlet oxygen as the cytotoxic agent in the photoactivation of a murine tumour. *Cancer Res.*, **36**, 2326–2329.

WHEATER PR, BURKITT HG AND DANIELS VG. (1979). Gastrointestinal system. In *The Functional Histology*. pp. 182–195. Churchill Livingstone: Edinburgh.